# The Boseman Effect: A Missed Opportunity?

**DOI:** 10.7759/cureus.24959

**Published:** 2022-05-13

**Authors:** Aaron Kahlam, Salil Chowdhury, Jasneel Kahlam, Kamal Amer, Sushil Ahlawat

**Affiliations:** 1 Internal Medicine, Rutgers New Jersey Medical School, Newark, USA; 2 Internal Medicine, New York Institute of Technology College of Osteopathic Medicine, Old Westbury, USA; 3 Gastroenterology and Hepatology, Rutgers New Jersey Medical School, Newark, USA

**Keywords:** public health, patient education (topic), internet use, colonic neoplasms, early detection of cancer

## Abstract

Introduction

Public figures, namely celebrities, are highly influential people whose actions and thoughts are often emulated, especially regarding healthcare. Understanding trends in public interest may provide an opportunity for further patient education. Given the changes of the COVID-19 pandemic along with the highly publicized death of actor Chadwick Boseman, who died from complications of colon cancer, we analyzed trends in colon cancer searches over a 15-month period.

Methods

Google Trends (Google, Mountain View, California) was used to access search histories in the United States from January 1, 2020, through April 30, 2021. Four search terms were analyzed: “colon cancer”, “colonoscopy”, “Cologuard”, and “virtual colonoscopy”. Google Trends reports data as relative search volume (RSV), a scaled number from 0-100 reflecting interest in a particular search term over a set time. Search terms were analyzed on the same RSV scale with one-way ANOVAs comparing search volumes during four eight-week blocks.

Results

Google Trends data was reported weekly. Search volume for colon cancer was higher (17.3, p=0.00) over the eight weeks following Boseman’s death, while search volume for colonoscopy returned to normal (21.5, p=0.95) when compared to pre-pandemic levels.

Conclusion

The peak in colon cancer searches in late August of 2020 corresponds to the death of Chadwick Boseman on 8/28/2020. Colonoscopy interest decreased during the COVID-19 pandemic before returning to previous levels around the time of Boseman’s death without experiencing the same spike in interest. This discrepancy represents a missed opportunity for patient education on this preventable disease.

## Introduction

With the rise of the coronavirus disease 2019 (COVID-19) pandemic and the ensuing restrictions that were placed, colon cancer screening has been significantly affected [1-3]. Given these new restrictions, interest in colon cancer, colon cancer screening, as well as other modalities for screening declined during the COVID-19 pandemic [4, 5]. This trend is concerning as colon cancer is the third most common cancer in the world and is the second most common cause of cancer-related death in the world [6]. It is thus essential that practitioners assess for ways to increase interest as well as garner attention to the importance of colon cancer screening. One such modality relies on the use of social media, namely celebrities.

Prior to the COVID-19 pandemic, interest in diseases, treatments, and prevention had been influenced by public figures and current events. Public figures are highly influential people who affect the general population's interest in a disease through either endorsement of medications, research, or personal experiences [7]. This is particularly true in the case of colon cancer screening. In the early 2000s, journalist Katie Couric lost her husband to colon cancer at the age of 42. Subsequently, she went on a campaign to raise awareness about colon cancer screening, leading to a significant increase in colonoscopies [8, 9]. In 2010, President Barack Obama underwent a virtual colonoscopy, leading to a statement from the American College of Gastroenterology [10, 11]. More recently, actor Chadwick Boseman died on August 28th, 2020, of colon cancer at the age of 43. While it may be too soon to see the long-term effects of Boseman’s death on colon cancer screening rates, recent research has shown that interest in colon cancer increased surrounding his death, particularly among Black Americans [12]. However, it is unclear if this interest in the disease translated into an interest in colon cancer screening and how long that effect might have lasted.

Our aim in this study is to analyze public interest in colon cancer and colon cancer screening using search volumes in the time surrounding Chadwick Boseman’s death. Specifically, we look to compare interest in colon cancer after Boseman’s death to the time immediately prior, as well as the time prior to the pandemic, to understand the overall impact of his death in the context of the COVID-19 pandemic.

This work was presented at the American College of Gastroenterology (ACG) Conference virtually on October 26th, 2021.

## Materials and methods

We performed an observational study using data from Google Trends (Google, Mountain View, California). Google Trends (GT) is a publicly available data set that reports relative search volume (RSV) by week for a given search term over a given timeframe or for a specific geographic location. RSV is a scaled value from 0 to 100 that reflects the frequency of a search for a particular term, with 100 being the highest search volume over a specific time period. The RSV value for a given term can change by changing the time period or changing the scale (i.e., comparing multiple terms on one scale). In this study, four search terms were analyzed on the same scale to allow for direct comparisons to be made. The terms queried were “colon cancer”, “colonoscopy”, “virtual colonoscopy” and “Cologuard”. RSV data was recorded by week in the United States from January 1, 2020, to April 30, 2021, for each search term. For the purposes of this study, data from other countries was excluded. The weekly RSV values were averaged for each search term, and these averages were compared using a one-way analysis of variance (ANOVA).

Next, four eight-week blocks were chosen to represent relevant periods during the study: 1/5/2020 - 2/23/2020 represented the pre-pandemic period in the United States, 6/28/2020 - 8/16/2020 represented the time immediately prior to Boseman’s death, 9/6/2020 - 11/22/2020 represented the period immediately after Boseman’s death, and 3/7/2021-4/25/2021 represented six months following Boseman’s death. Due to the significant changes in RSV in the week of and the week following Boseman’s death, these two weeks were excluded from this analysis. The eight RSV values for each block were averaged for each search term. The pre-pandemic period was set as the reference, and another one-way ANOVA was performed to compare RSV for each search term during these relevant timeframes. All statistical analysis was performed using SPSS version 24 (IBM Inc., Armonk, New York).

## Results

**Figure 1:**
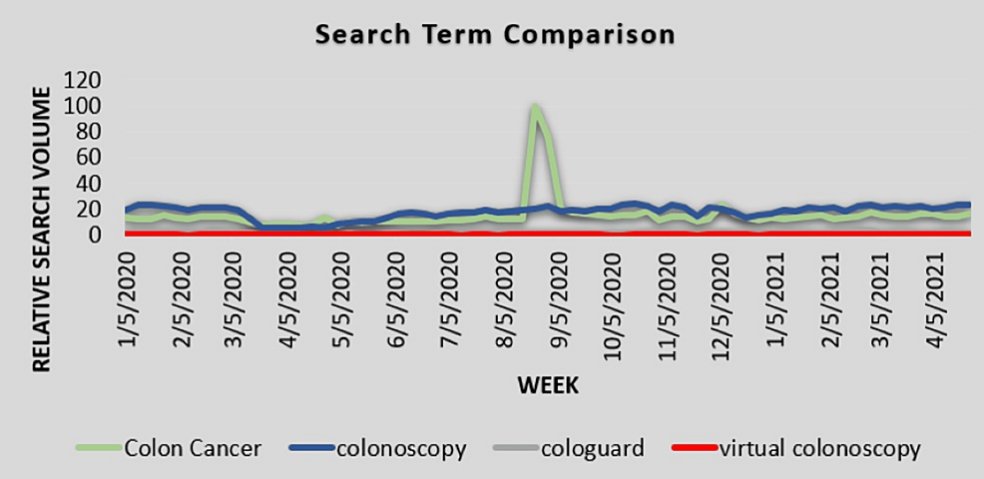
Search term comparison Comparison graph of the relative search volume for all four search terms.

RSVs for the search terms “colon cancer”, “colonoscopy”, “virtual colonoscopy”, and “Cologuard” are shown by week in Figure 1. Between the week starting on 8/16/2020 and the week starting on 8/23/2020, there was a 669% increase in searches for the term “colon cancer”, likely corresponding with Boseman’s death on 8/28/2020. However, in the same week, searches for the term “colonoscopy” rose 5%, and searches for “Cologuard” and “virtual colonoscopy” stayed the same compared to the week prior. In the week following, RSV for “colon cancer” dropped 24% from its high before dropping another 72% in the subsequent week, close to its original baseline prior to Boseman’s death (Figure 1).

Results from the one-way ANOVA comparing the overall RSV from 1/1/2020 through 4/30/2021 between the search terms are shown in Table 1. The term “colonoscopy” had the highest RSV, although this was not significantly different from “colon cancer”. Table 2 shows the results of the one-way ANOVA comparing RSV in the four different eight-week periods. RSV for the search term “colon cancer” was higher during the time following Boseman’s death. However, by six months, RSV had returned to its pre-pandemic levels. The search term “colonoscopy” had a lower RSV in the time just prior to Boseman’s death. Following his death, RSV returned to its pre-pandemic levels and stayed the same six months out. Both “Cologuard” and “virtual colonoscopy” did not have significant differences in their search volume over the four time periods.

**Table 1:** RSV comparison between search terms Results of ANOVA comparing average RSV of search terms from 1/1/2020 - 4/30/2021. The search term "colon cancer" was used as the reference (ref) in this comparison. RSV - relative search volume

Search term	RSV + SD	p-value
Colon cancer	15.9 + 13.1	Ref
Colonoscopy	18.5 + 5.2	0.21
Cologuard	1.9 + 0.7	0.00
Virtual colonoscopy	0.4 + 0.2	0.00

**Table 2:** RSV comparison for search terms over four eight-week blocks Results of ANOVA comparing average RSV of each search term for eight-week periods prior to the pandemic, immediately prior to Boseman’s death, immediately following Boseman’s death, and six months following Boseman’s death. The pre-pandemic average for each search term was used as the reference (ref) for this analysis, and thus do not have an associated p-value. RSV - relative search volume

Search term	Average RSV (p-value) 1/5/20 - 2/23/20	Average RSV (p-value) 6/28/20 - 8/16/20	Average RSV (p-value) 9/6/20 - 11/22/20	Average RSV (p-value) 3/7/21 - 4/25/21
Colon cancer	14.1 (ref)	12.8 (0.28)	17.3 (0.00)	15.9 (0.11)
Colonoscopy	22.1 (ref)	18.1 (0.00)	21.5 (0.91)	22.63 (0.95)
Cologuard	2.4 (ref)	1.8 (0.22)	2.0 (0.64)	2.4 (1.00)
Virtual colonoscopy	0.44 (ref)	0.38 (0.93)	0.38 (0.93)	0.0 (0.93)

## Discussion

In this study, we examined how the death of Chadwick Boseman influenced interest in colon cancer and its prevention as measured by search volume online through GT. We found that while his death certainly influenced interest in colon cancer, it did not lead to increased interest in screening for colon cancer. Interestingly, we found that despite the spike in interest for colon cancer, the average interest in colonoscopy was higher overall, though this difference was not significant. Search volume for colonoscopies had declined sharply in the early stages of the pandemic and only returned to pre-pandemic levels around the time of Boseman’s death. Interest in colonoscopies did not experience the same spike that interest in colon cancer did. Furthermore, despite being a potentially useful option for screening during the pandemic, interest in stool-based tests such as Cologuard remained low throughout. Overall, these results represent a missed opportunity to remind the public about important health screening beyond preventing COVID-19. 

Our results are consistent with a prior study showing a decrease in searches related to colonoscopies around March 2020 [4]. Furthermore, a study using data from the Cancer Research Network showed an 84.5% decrease in colorectal screening during April 2020 [13], while another study by the Epic Health Research Network showed a decrease in breast and colon cancer screening of about 90% in the early months of the pandemic [13], supporting our hypothesis that decreased search volume correlated to decreased screening. The decrease in screening was likely related to the fact that elective procedures were banned as the COVID-19 pandemic worsened in the United States around March 2020. Another study similarly showed a spike in colon cancer searches around Boseman’s death in late August 2020 [12]. However, in our study, search volume for “colon cancer” and “colonoscopy” returned to their pre-pandemic levels by April 2021, which is contrary to the influence celebrities have had in the past. For example, when Katie Couric lost her husband to colon cancer, she went on a campaign in the early 2000s that led to an increase in colonoscopies [8, 9]. Going as far as televising her colonoscopy on the Today Show, she helped take away some of the embarrassment that patients might have felt discussing the invasive, uncomfortable procedure [14]. Another prominent example of celebrity influence on public health was when Angelina Jolie announced she was a BRCA1 mutation carrier, leading her to undergo a double mastectomy [15, 16]. Interestingly, while Katie Couric influenced colon cancer screening, breast cancer screening rates were unchanged after Angelina Jolie’s announcement [17]. Instead, interest in prophylactic mastectomies increased significantly [18].

Unfortunately, our results show that the effect of Chadwick Boseman’s death on colon cancer awareness was transient. One reason for this may be the COVID-19 pandemic that was still drawing the attention of the public and health experts alike. As the pandemic waxed and waned, it is likely that increased cases in the fall drew interest back to COVID-19 and symptoms related to it [5, 19]. Additionally, with news of vaccines coming in late 2020, searches related to the vaccine increased, drawing additional attention to the pandemic [20]. With an overlying health crisis shifting focus away from colon cancer, it was difficult to assess how much an effect Boseman’s death could have had. Additionally, Boseman was more private about his life, including his diagnosis of stage III colon cancer in 2016 [21]. While Jolie and Couric were in the public eye leading campaigns to raise awareness, Boseman and his family kept to themselves. A more analogous comparison would be with senator John McCain, who was diagnosed with glioblastoma. Internet interest around brain tumors spiked in the two weeks surrounding his death before quickly returning to baseline, as seen with Boseman [22]. It is likely that after the immediate shock, people slowly returned to their daily lives and the focus shifted away from Boseman and colon cancer. The decreasing interest in colon cancer after Boseman’s diagnosis and death, combined with the news surrounding the COVID-19 pandemic along with other possible factors likely led to the missed opportunity to further educate the public on this important disease.

There are some important limitations in our study to address. First, internet interest is only a surrogate measure for public interest. It is difficult to know who searched for the terms in this study and how many times one person may have searched a specific term. Additionally, it will take time to determine the actual effect on colon cancer screening rates that Boseman’s death had. Our results also cannot speculate on the quality of information people searching these terms encountered and what they may have done with that information. For example, it is likely people were searching for colon cancer as a cause of death and not out of interest in learning more about the disease. Finally, we only looked at four search terms, and it would be difficult to cover all possible terms, including potential misspellings, that the public could search in relation to this topic.

## Conclusions

In conclusion, we show in this study that interest in colon cancer spiked in relation to Chadwick Boseman’s death on August 28th, 2020. However, this effect was transient, and by April 2021, RSV for colon cancer had returned to its pre-pandemic baseline. Additionally, while colon cancer drew more interest, three prominent colon cancer screening options did not draw any additional interest in the same way. As the world attempts to control the spread of COVID-19 and return to life prior to the pandemic, it will be important to continue promoting screening for colon cancer and reduce mortality in diseases other than COVID-19.
